# Canagliflozin protects against cisplatin-induced acute kidney injury by AMPK-mediated autophagy in renal proximal tubular cells

**DOI:** 10.1038/s41420-021-00801-9

**Published:** 2022-01-10

**Authors:** Cheol Ho Park, Bin Lee, Myeonggil Han, Woo Joong Rhee, Man Sup Kwak, Tae-Hyun Yoo, Jeon-Soo Shin

**Affiliations:** 1 Department of Microbiology, Yonsei University College of Medicine, Seoul, Republic of Korea; 2 Department of Internal Medicine, Institute of Kidney Disease Research, Yonsei University College of Medicine, Seoul, Republic of Korea; 3 Institute for Immunology and Immunological Diseases, Yonsei University College of Medicine, Seoul, Republic of Korea; 4 Brain Korea 21 FOUR Project for Medical Science, Yonsei University College of Medicine, Seoul, Republic of Korea; 5 Severance Biomedical Science Institute, Yonsei University College of Medicine, Seoul, Republic of Korea

**Keywords:** Acute kidney injury, Apoptosis

## Abstract

Sodium-glucose cotransporter 2 inhibitors, which are recently introduced as glucose-lowering agents, improve cardiovascular and renal outcomes in patients with diabetes mellitus. These drugs also have beneficial effects in various kidney disease models. However, the effect of SGLT2 inhibitors on cisplatin-induced acute kidney injury (AKI) and their mechanism of action need to be elucidated. In this study, we investigated whether canagliflozin protects against cisplatin-induced AKI, depending on adenosine monophosphate-activated protein kinase (AMPK) activation and following induction of autophagy. In the experiments using the HK-2 cell line, cell viability assay and molecular analysis revealed that canagliflozin protected renal proximal tubular cells from cisplatin, whereas addition of chloroquine or compound C abolished the protective effect of canagliflozin. In the mouse model of cisplatin-induced AKI, canagliflozin protected mice from cisplatin-induced AKI. However, treatment with chloroquine or compound C in addition to administration of cisplatin and canagliflozin eliminated the protective effect of canagliflozin. Collectively, these findings indicate that canagliflozin protects against cisplatin-induced AKI by activating AMPK and autophagy in renal proximal tubular cells.

## Introduction

Sodium-glucose cotransporter 2 (SGLT2) inhibitors not only showed glucose-lowering effects but also showed unexpected cardioprotective and renoprotective effects in patients with type 2 diabetes mellitus in large clinical trials [1–6]. Recent clinical trials showed that even non-diabetic patients experience the benefits of SGLT2 inhibitors; preclinical studies that use non-diabetic kidney disease models, such as renal ischemia-reperfusion injury (IRI) model, proteinuric non-diabetic nephropathy model, and renal injury in prediabetic rat models, revealed the protective effect of SGLT2 inhibitors [7–12]. The evidence that the renoprotective effect of SGLT2 inhibitors occurs regardless of the glucose-lowering properties supports the rationale for testing the therapeutic use of the drugs to treat other kidney diseases.

Cisplatin, a chemotherapeutic agent approved by the United States Food and Drug Administration in 1978, is used in the treatment of various solid organ malignancies [13]. However, ~30% of patients treated with cisplatin develop acute kidney injury (AKI), and nephrotoxicity has been accepted as the most dose-limiting toxicity [14]. Cisplatin-induced AKI has been one of the most frequently used mouse models for tubulotoxic AKI with its simplicity, reproducibility, and clinical relevance [15]. In the model of cisplatin-induced AKI, cisplatin affects the proximal tubular cells and induces cell death by damaging DNA, mitochondria, and other cellular organelles [16]. Although many studies using the mouse model of cisplatin-induced AKI to test therapeutic measures for tubulotoxic AKI have been conducted, the effects of renoprotective strategies derived from those studies are mostly partial or limited [14].

Autophagy, a highly conserved protein degradation pathway in eukaryotic cells, plays a vital role for cellular homeostasis [17]. Since the kidney, especially the proximal tubule, consumes a large amount of energy due to its functions, it has been assumed that autophagy plays an important role in the physiology and homeostasis maintenance of the proximal tubule [18]. Recent studies have confirmed this assumption that describes the cytoprotective role of autophagy in various pathological conditions including cisplatin-induced AKI [19–23].

Many studies have attempted to explain the underlying mechanisms of unexpected cardiovascular and renal benefits elicited by SGLT2 inhibitors, and autophagy has been suggested as one of those mechanisms [24, 25]. However, the protective effect of SGLT2 inhibitors against cisplatin-induced AKI has rarely been examined, and how SGLT2 inhibitors could induce this protective effect is unknown. Therefore, this study aims to test the hypothesis that SGLT2 inhibitors protect against cisplatin-induced AKI and that AMPK-mediated autophagy is an underlying mechanism.

## Results

### Canagliflozin protects HK-2 cells from cisplatin by inhibiting apoptosis

**Fig. 1:**
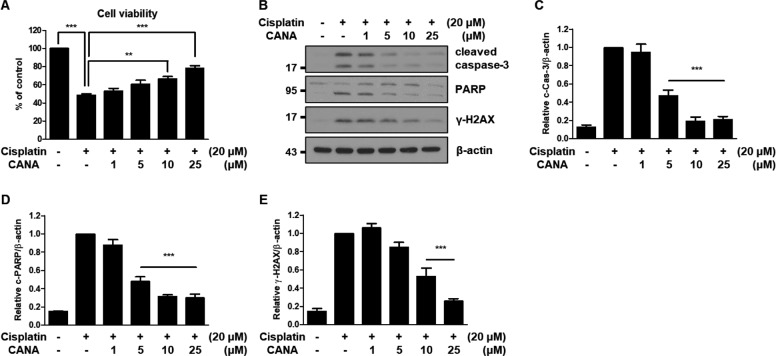
Canagliflozin protects HK-2 cells from cisplatin by inhibiting apoptosis. Human renal proximal tubular (HK-2) cells were treated with 20 μM cisplatin in the absence or presence of canagliflozin (CANA) at the indicated concentrations for 24 h. **A** Cell viability was evaluated via CCK-8 assay. Data are expressed as mean ± SD (*N* = 4). Differences between groups were evaluated using one-way ANOVA followed by Bonferroni’s multiple comparison test. **P < 0.01, ***P < 0.001, significantly different from the cisplatin-only treated group. **B** Representative immunoblot analysis. **C**–**E** Densitometric analysis of immunoblots to estimate the relative abundance of indicated molecules as normalized to that of β-actin. Data are expressed as mean ± SD (*N* = 3). Differences between groups were evaluated using one-way ANOVA followed by Bonferroni’s multiple comparison test. ***P < 0.001, significantly different from the cisplatin-only treated group.

To test whether canagliflozin protects HK-2 cells from cisplatin-induced cytotoxicity, HK-2 cells were treated with 20 μM cisplatin and 1–25 μM canagliflozin for 24 h because treatment with 20 μM cisplatin for 24 h showed 50% cell viability compared to the control and a pharmacokinetics study revealed that the daily approved dose of canagliflozin shows a peak plasma concentration of 10 μM in humans and concentrations of canagliflozin did not affect cell viability compared to the control (Supplementary Fig. 1A–C) [26]. The cell viability assay showed that canagliflozin attenuated cisplatin-induced cytotoxicity in a concentration-dependent manner in HK-2 cells (Fig. 1A). Quantitatively, cisplatin-only treated cells showed 50% cell viability compared to the control, and cells treated with cisplatin + 25 μM canagliflozin showed 80% cell viability compared to the control. These results were further validated by immunoblot analysis. In accordance with the cell viability assay, canagliflozin treatment decreased the expression of various apoptosis markers, such as cleaved caspase-3, cleaved PARP, and γ-H2AX, compared to the cisplatin-only treated group (Fig. 1B–E). These results suggest that canagliflozin protects HK-2 cells from cisplatin-induced cytotoxicity by inhibiting apoptosis. We also evaluated whether other SGLT2 inhibitors protect HK-2 cells from cisplatin. In contrast to canagliflozin, dapagliflozin, and empagliflozin failed to attenuate cisplatin-induced cytotoxicity in HK-2 cells (Supplementary Fig. 1D, E).

### Canagliflozin induces autophagy in HK-2 cells and protects HK-2 cells from cisplatin in an autophagy-dependent manner

**Fig. 2:**
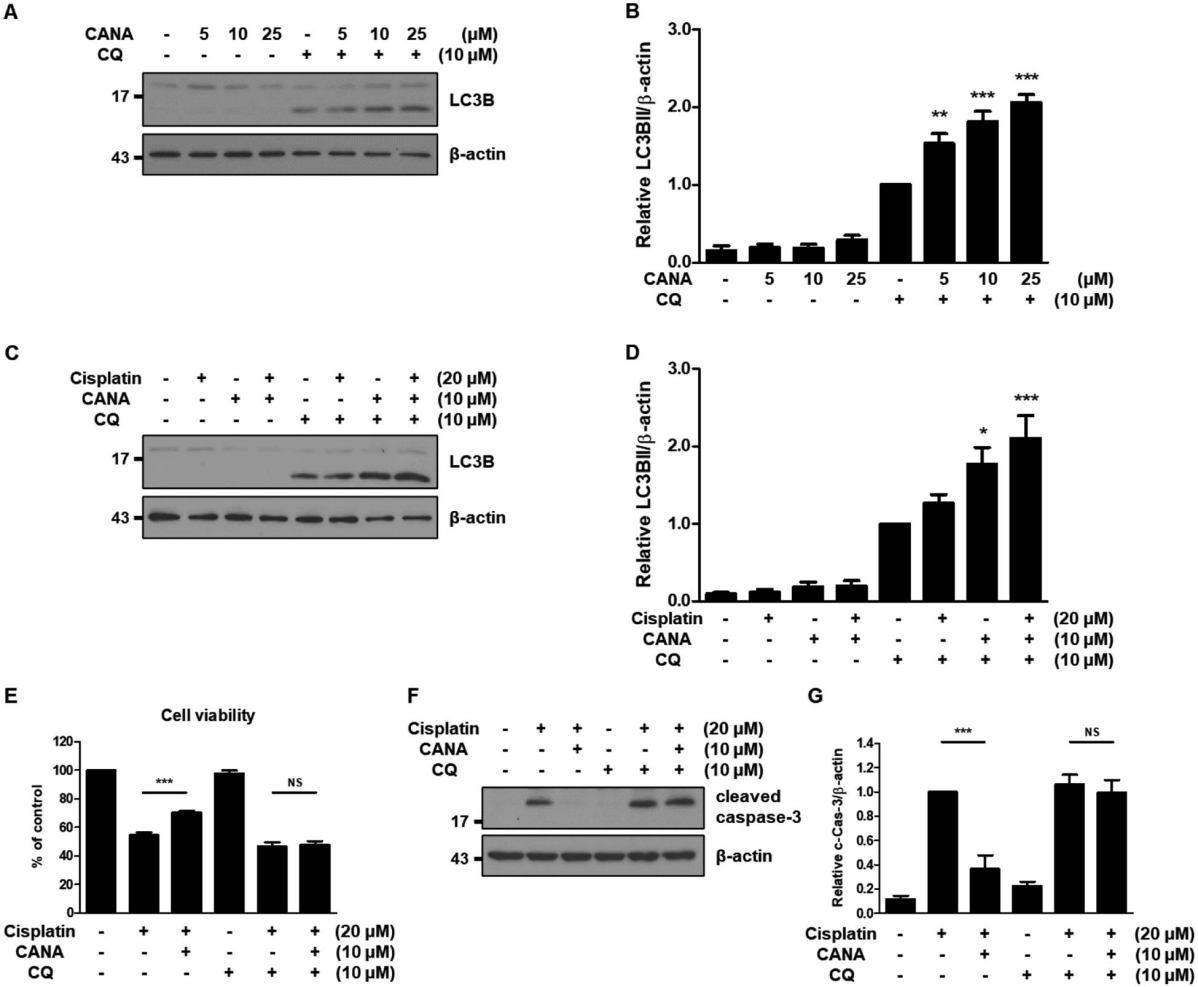
Canagliflozin induces autophagy in HK-2 cells and protects HK-2 cells from cisplatin in an autophagy-dependent manner. HK-2 cells were treated with the indicated concentrations of canagliflozin (CANA) in the absence or presence of 10 μM chloroquine (CQ) for 24 h (**A**–**B**). **A** Representative immunoblot analysis. **B** Densitometric analysis of immunoblots to estimate the relative abundance of LC3BII normalized to that of β-actin. Data are expressed as mean ± SD (*N* = 5). Differences between groups were evaluated using one-way ANOVA followed by Bonferroni’s multiple comparison test. **P < 0.01, ***P < 0.001, significantly different from the chloroquine-only treated group. HK-2 cells were treated with indicated concentrations of cisplatin, canagliflozin, and/or chloroquine for 24 h (**C**–**G).** **C** Representative immunoblot analysis. **D** Densitometric analysis of immunoblots to estimate the relative abundance of LC3BII normalized to that of β-actin. Data are expressed as mean ± SD (*N* = 5). Differences between groups were evaluated using one-way ANOVA followed by Bonferroni’s multiple comparison test. *P < 0.05, ***P < 0.001, significantly different from the chloroquine-only treated group. **E** Cell viability was evaluated via CCK-8 assay. Data are expressed as mean ± SD (*N* = 6). Differences between groups were evaluated using one-way ANOVA followed by Bonferroni’s multiple comparison test. ***P < 0.001. NS, not significant. **F** Representative immunoblot analysis. **G** Densitometric analysis of immunoblots to estimate the relative abundance of cleaved caspase-3 as normalized that of β-actin. Data are expressed as mean ± SD (*N* = 4). Differences between groups were evaluated using one-way ANOVA followed by Bonferroni’s multiple comparison test. ***P < 0.001. NS, not significant.

We examined whether canagliflozin induces autophagy in HK-2 cells. We treated HK-2 cells with canagliflozin at 5–25 μM in the absence or presence of chloroquine for 24 h to measure the autophagic flux. Immunoblot analysis of LC3B showed that canagliflozin induces autophagy in a concentration-dependent manner (Fig. 2A, Supplementary Fig. 2A). In the densitometric analysis, the relative abundance of LC3BII exhibited a 1.53-fold, 1.82-fold, and 2.06-fold increase in 5, 10, and 25 μM canagliflozin-treated HK-2 cells in the presence of chloroquine, respectively, compared to the chloroquine-only treated HK-2 cells (Fig. 2B). Next, we examined the effect of canagliflozin on cisplatin-treated HK-2 cells in terms of autophagy. HK-2 cells were treated with cisplatin and/or canagliflozin in the absence or presence of chloroquine. Immunoblot analysis of LC3B revealed that co-treatment with cisplatin and canagliflozin further induced autophagy compared to the treatment of HK-2 cells with cisplatin (Fig. 2C, Supplementary Fig. 2B). Quantitatively, the relative abundance of LC3BII showed a 1.26-fold increase in cisplatin-treated cells, 1.77-fold in canagliflozin-treated cells, and 2.10-fold in cisplatin-canagliflozin-treated cells in the presence of chloroquine compared to the HK-2 cells treated only with chloroquine (Fig. 2D).

To investigate the role of autophagy in the protective effect of canagliflozin, we tested the effect of canagliflozin on the cisplatin-induced cytotoxicity or apoptosis of HK-2 cells in the absence or presence of chloroquine. The cell viability assay showed that the protective effect of canagliflozin against cisplatin-induced cytotoxicity disappeared in the presence of chloroquine, although canagliflozin treatment with chloroquine did not affect the viability of HK-2 cells (Fig. 2E, Supplementary Fig. 2C). In the absence of chloroquine, cisplatin treatment led to ~55% cell viability, which increased to 70% with canagliflozin treatment. However, in the presence of chloroquine, cisplatin and canagliflozin treatments showed similar values of cell viability (47 and 48%, respectively). This observation was largely maintained in the immunoblot analysis of cleaved caspase-3 and Annexin V/PI staining (Fig. 2F, G, Supplementary Fig. 2D–F). Similarly, treatment with another autophagy inhibitor, bafilomycin A, replicated these observations (Supplementary Fig. 2G–J).

### Canagliflozin activates AMPK and inhibits mTOR in HK-2 cells and protects HK-2 cells from cisplatin in an AMPK activation-dependent manner

**Fig. 3:**
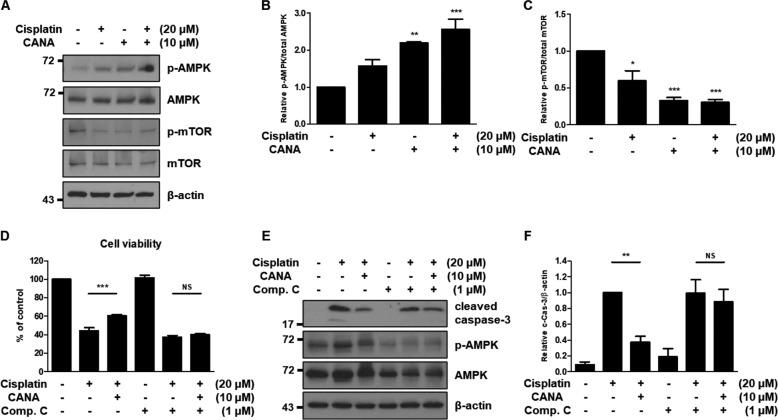
Canagliflozin activates AMPK and inhibits mTOR in HK-2 cells and protects HK-2 cells from cisplatin in an AMPK activation-dependent manner. HK-2 cells were treated with indicated concentrations of cisplatin and/or canagliflozin (CANA) for 24 h (**A**–**C**). **A** Representative immunoblot analysis. **B**, **C** Densitometric analysis of immunoblots to estimate the relative abundance of indicated molecules as normalized that of total AMPK or total mTOR, respectively. Data are expressed as mean ± SD (*N* = 4). Differences between groups were evaluated one-way ANOVA followed by Bonferroni’s multiple comparison test. *P < 0.05, **P < 0.01, ***P < 0.001, significantly different from the control. HK-2 cells were treated with indicated concentrations of cisplatin, canagliflozin, and/or compound C (Comp. C) for 24 h (**D**–**F**). **D** Cell viability was evaluated by CCK-8 assay. Data are expressed as mean ± SD (N = 5). Differences between groups were evaluated using one-way ANOVA followed by Bonferroni’s multiple comparison test. ***P < 0.001. NS, not significant. **E** Representative immunoblot analysis. **F** Densitometric analysis of immunoblots to estimate the relative abundance of cleaved caspase-3 as normalized that of β-actin. Data are expressed as mean ± SD (*N* = 5). Differences between groups were evaluated using one-way ANOVA followed by Bonferroni’s multiple comparison test. **P < 0.01. NS, not significant.

Since the AMPK-mTOR pathway is a major upstream signaling pathway that regulates autophagy, we examined whether canagliflozin activates AMPK and inactivates mTOR in HK-2 cells and protects HK-2 cells from cisplatin in an AMPK activation-dependent manner using compound C, an AMPK inhibitor [27]. In the experiment, canagliflozin treatment upregulated AMPK phosphorylation and downregulated mTOR phosphorylation (Fig. 3A). Densitometric analyses revealed that treatment with 20 μM cisplatin and 10 μM canagliflozin upregulated AMPK phosphorylation by 2.56-fold and downregulated mTOR phosphorylation by 0.30-fold compared to the control, respectively (Fig. 3B, C). The cell viability assay using cisplatin, canagliflozin, and/or compound C showed that the protective effect of canagliflozin against cisplatin-induced cytotoxicity was abolished with compound C treatment, although compound C treatment with canagliflozin did not affect the viability of HK-2 cells (Fig. 3D, Supplementary Fig. 3A). Without compound C treatment, cisplatin treatment resulted in 44% cell viability, which increased to 61% in the presence of canagliflozin. With compound C treatment, cisplatin treatment led to 37% cell viability, which was not significantly changed (40%) with canagliflozin treatment. The immunoblot analysis of cleaved caspase-3 showed that treatment with compound C eliminated the protective effect of canagliflozin from cisplatin-induced cytotoxicity in HK-2 cells (Fig. 3E, F).

### Canagliflozin attenuates cisplatin-induced AKI and increases autophagy in mice

**Fig. 4:**
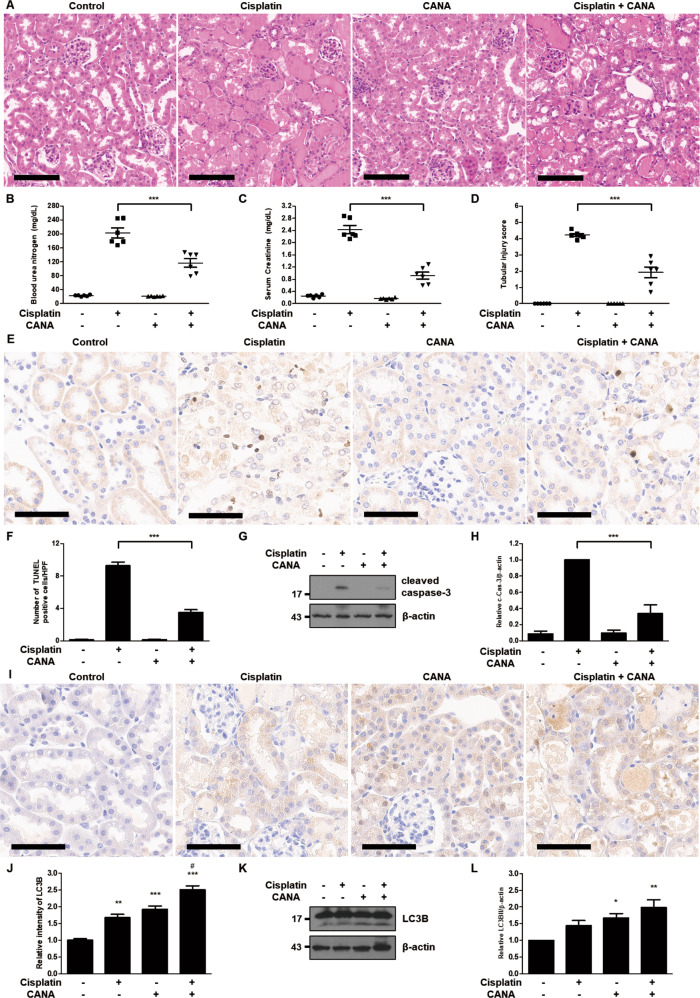
Canagliflozin attenuates cisplatin-induced AKI and increases autophagy in mice. C57BL/6 mice were administered with canagliflozin (CANA) (10 mg/kg) or vehicle orally for five consecutive days, and intraperitoneal injection of cisplatin (20 mg/kg) or saline was done on the 5th day of the treatment. Mice were euthanized at 72 h after cisplatin injection to collect blood samples for measurements of blood urea nitrogen and serum creatinine and kidney tissues for histology. **A** Representative images of kidney H–E staining (scale bar = 100 μm). **B** Blood urea nitrogen. **C** Serum creatinine. **D** Tubular injury score. Data are expressed as mean ± SD (*N* = 6). Differences between groups were evaluated using one-way ANOVA followed by Bonferroni’s multiple comparison test. ***P < 0.001. **E** Representative images of TUNEL staining (scale bar = 60 μm). **F** Quantification of TUNEL-positive cells in kidney tissues. Data are expressed as mean ± SD (*N* = 6). Differences between groups were evaluated using one-way ANOVA followed by Bonferroni’s multiple comparison test. ***P < 0.001. **G** Representative immunoblot analysis. **H** Densitometric analysis of immunoblots to estimate the relative abundance of cleaved caspase-3 as normalized that of β-actin. Data are expressed as mean ± SD (*N* = 6). Differences between groups were evaluated using one-way ANOVA followed by Bonferroni’s multiple comparison test. ***P < 0.001. **I** Representative images of IHC staining of LC3B (scale bar = 60 μm). **J** Quantitative analysis of IHC staining. Data are expressed as mean ± SD (*N* = 6). Differences between groups were evaluated using one-way ANOVA followed by Bonferroni’s multiple comparison test. **P < 0.01, ***P < 0.001, significantly different from the control. ^#^*P* < 0.001, significantly different from the cisplatin-only treated group. **K** Representative immunoblot analysis. **L** Densitometric analysis of immunoblots to estimate the relative abundance of LC3BII as normalized that of β-actin. Data are expressed as mean ± SD (*N* = 6). Differences between groups were evaluated using one-way ANOVA followed by Bonferroni’s multiple comparison test. *P < 0.05, **P < 0.01, significantly different from the control.

We investigated the effects of canagliflozin in the mouse model of cisplatin-induced AKI. Cisplatin treatment led to extensive tubular damage, and administration of canagliflozin together with cisplatin treatment attenuated the severity of tubular damage as indicated in H–E staining results (Fig. 4A). Consistently, canagliflozin treatment suppressed BUN and serum creatinine elevation from 245 to 147 mg/dL and from 2.8 to 1.3 mg/dL, respectively, in cisplatin-treated mice (Fig. 4B, C). The tubular injury score showed a similar pattern: a decrease from 4.6 in the cisplatin-only treated group to 2.9 in the cisplatin-canagliflozin-treated group (Fig. 4D). TUNEL staining results revealed that canagliflozin decreased the number of TUNEL-positive cells in the kidneys of cisplatin-treated mice (Fig. 4E, F). Cisplatin treatment also induced apoptosis in mice kidneys as indicated by cleaved caspase-3, which was ameliorated via canagliflozin administration (Fig. 4G, H). We also examined the effect of canagliflozin on tubular autophagy in the mouse model of cisplatin-induced AKI. In the immunohistochemistry staining of mice kidneys, both cisplatin-only treatment and canagliflozin-only treatment enhanced LC3B staining, and canagliflozin treatment further enhanced LC3B staining in cisplatin-treated mice kidneys (Fig. 4I). In the quantitative analysis of LC3B staining intensity, the relative intensity of LC3B showed a 1.67-fold increase in the cisplatin-treated group, 1.91-fold in the canagliflozin-treated group, and 2.50-fold in the cisplatin-canagliflozin-treated group compared to the control group (Fig. 4J). Similarly, the immunoblot analysis of LC3B demonstrated that canagliflozin treatment enhances the expression of LC3BII in mice kidneys (Fig. 4K, L).

### Inhibition of autophagy eliminates the protective effect of canagliflozin against cisplatin-induced AKI in mice

**Fig. 5:**
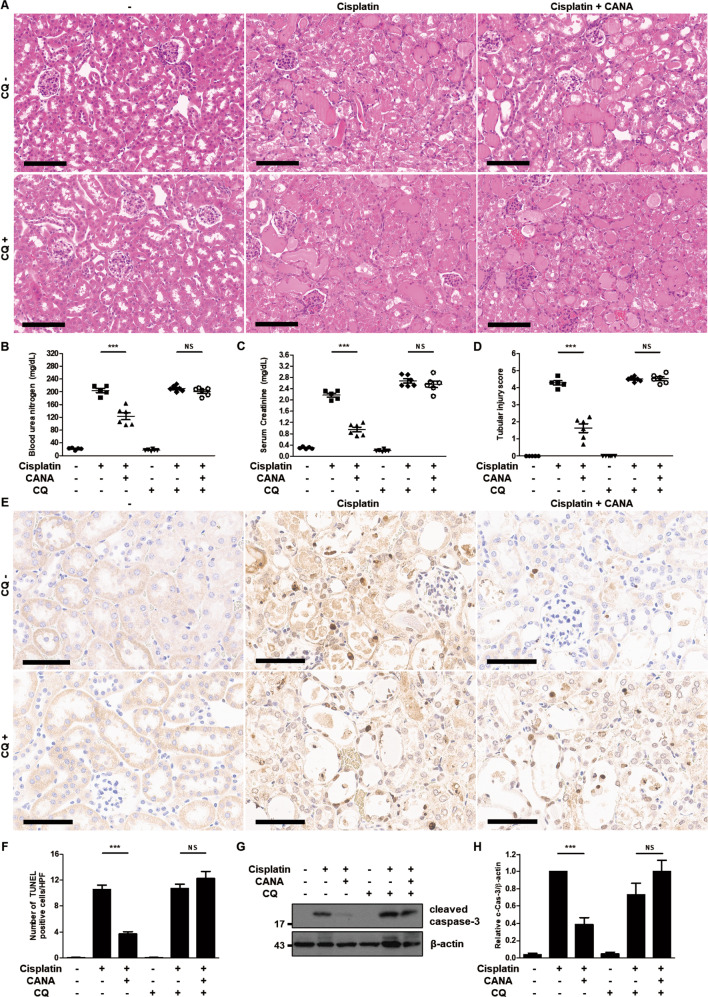
Inhibition of autophagy eliminates the protective effect of canagliflozin against cisplatin-induced AKI in mice. C57BL/6 mice were administered with canagliflozin (CANA) (10 mg/kg) or vehicle orally and intraperitoneally injected with chloroquine (CQ) (60 mg/kg) or saline for five consecutive days, and intraperitoneal injection of cisplatin (20 mg/kg) or saline was done on the 5th day of the treatment. Mice were euthanized at 72 h after cisplatin injection to collect blood samples for measurements of blood urea nitrogen and serum creatinine and kidney tissues for histology. **A** Representative images of kidney H–E staining (scale bar = 100 μm). **B** Blood urea nitrogen. **C** Serum creatinine. **D** Tubular injury score. Data are expressed as mean ± SD (*N* = 5–6). Differences between groups were evaluated using one-way ANOVA followed by Bonferroni’s multiple comparison test. ***P < 0.001. NS, not significant. **E** Representative images of TUNEL staining (scale bar = 60 μm). **F** Quantification of TUNEL-positive cells in kidney tissues. Data are expressed as mean ± SD (*N* = 5–6). Differences between groups were evaluated using one-way ANOVA followed by Bonferroni’s multiple comparison test. ***P < 0.001. NS, not significant. **G** Representative immunoblot analysis. **H** Densitometric analysis of immunoblots to estimate the relative abundance of cleaved caspase-3 as normalized that of β-actin. Data are expressed as mean ± SD (*N* = 5). Differences between groups were evaluated using one-way ANOVA followed by Bonferroni’s multiple comparison test. ***P < 0.001. NS, not significant.

To determine the role of autophagy in the mouse model of cisplatin-induced AKI, we examined the role of chloroquine in the renoprotective effect of canagliflozin on cisplatin-treated mice. H–E staining results indicated that cisplatin treatment induced substantial tubular damage and canagliflozin-cisplatin treatment ameliorated the extent of tubular damage in the absence of chloroquine treatment. However, canagliflozin treatment failed to reduce tubular damage induced by cisplatin treatment in the presence of chloroquine (Fig. 5A). Canagliflozin treatment with chloroquine did not induce tubular damage (Supplementary Fig. 4A). In biochemical analysis, canagliflozin treatment reduced blood urea nitrogen (BUN) and serum creatinine elevation from 218 to 164 mg/dL and from 2.3 to 1.2 mg/dL, respectively, upon the cisplatin treatment in the absence of chloroquine. Although canagliflozin and chloroquine treatments did not elevate BUN or serum creatinine (Supplementary Fig. 4B, C), canagliflozin treatment showed comparable levels of BUN and serum creatinine: 224 to 212 mg/dL and 2.9 to 3.0 mg/dL, respectively, in the presence of chloroquine (Fig. 5B, C). Tubular injury score showed a similar pattern. Cisplatin-treated mice had a tubular injury score of 4.7 and cisplatin-canagliflozin-treated mice had a tubular injury score of 2.3 in the absence of chloroquine, whereas cisplatin-treated mice had a tubular injury score of 4.7 and cisplatin-canagliflozin-treated mice had a tubular injury score of 4.9 in the presence of chloroquine (Fig. 5D). In line with the biochemical and histological analysis, TUNEL staining showed that reduction in the number of TUNEL-positive cells induced by canagliflozin treatment was abolished when chloroquine treatment was added in the kidneys of cisplatin-treated mice (Fig. 5E, F). Immunoblot analysis of mice kidneys demonstrated that canagliflozin treatment reduced (did not reduce) the expression of cleaved caspase-3 induced by cisplatin administration without (with) chloroquine treatment (Fig. 5G, H).

### Inhibition of AMPK activation eliminates the protective effect of canagliflozin against cisplatin-induced AKI in mice

**Fig. 6:**
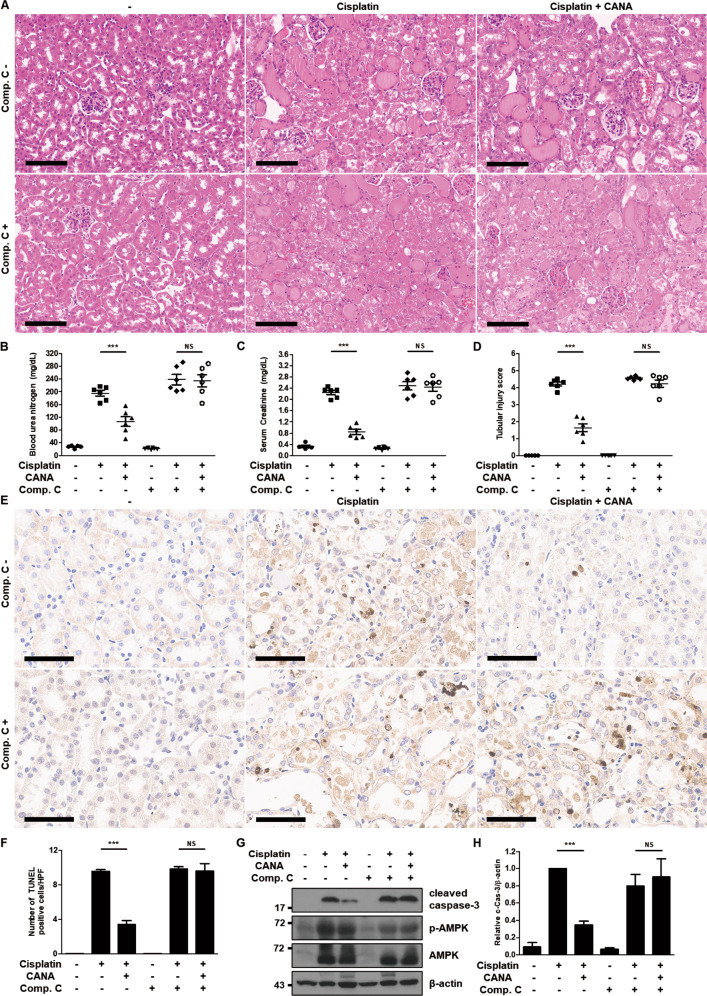
Inhibition of AMPK activation eliminates the protective effect of canagliflozin against cisplatin-induced AKI in mice. C57BL/6 mice were administered with canagliflozin (CANA) (10 mg/kg) or vehicle orally and intraperitoneally injected with compound C (Comp. C) (5 mg/kg) or saline for five consecutive days, and intraperitoneal injection of cisplatin (20 mg/kg) or saline was done on the 5th day of the treatment. Mice were euthanized at 72 h after cisplatin injection to collect blood samples for measurements of blood urea nitrogen and serum creatinine and kidney tissues for histology. **A** Representative images of kidney H–E staining (scale bar = 100 μm). **B** Blood urea nitrogen. **C** Serum creatinine. **D** Tubular injury score. Data are expressed as mean ± SD (*N* = 5–6). Differences between groups were evaluated using one-way ANOVA followed by Bonferroni’s multiple comparison test. ***P < 0.001. NS, not significant. **E** Representative images of TUNEL staining (scale bar = 60 μm). **F** Quantification of TUNEL-positive cells in kidney tissues. Data are expressed as mean ± SD (*N* = 5–6). Differences between groups were evaluated using one-way ANOVA followed by Bonferroni’s multiple comparison test. ***P < 0.001. NS, not significant. **G** Representative immunoblot analysis. **H** Densitometric analysis of immunoblots to estimate the relative abundance of cleaved caspase-3 as normalized that of β-actin. Data are expressed as mean ± SD (*N* = 5). Differences between groups were evaluated using one-way ANOVA followed by Bonferroni’s multiple comparison test. ***P < 0.001. NS, not significant.

To delineate whether canagliflozin exerts a renoprotective effect on the mouse model of cisplatin-induced AKI in an AMPK activation-dependent manner, we tested the role of compound C in the protective effect of canagliflozin on cisplatin-treated mice. H–E staining indicated that canagliflozin administration decreased (did not decrease) tubular damage induced by cisplatin treatment without (with) compound C treatment (Fig. 6A). Similar to the case of chloroquine treatment, administration of canagliflozin with compound C did not lead to tubular damage (Supplementary Fig. 5A). Canagliflozin treatment reduced BUN and serum creatinine from 217 to 156 mg/dL and from 2.5 to 1.2 mg/dL, respectively, in the cisplatin-administered mice without compound C treatment. Treatment with canagliflozin and compound C had no effect on BUN and serum creatinine (Supplementary Fig. 5B, C), and similarly canagliflozin did not have a significant impact on BUN and serum creatinine; the values changed from 293 to 288 mg/dL and from 3.0 to 2.9 mg/dL, respectively, in the cisplatin-administered mice with compound C treatment (Fig. 6B, C). The semiquantitative analysis demonstrated that canagliflozin-cisplatin treatment in the absence of compound C reduced the tubular injury score from 4.5 to 2.3, whereas it failed to reduce the score in the presence of compound C (the value remained at 4.7) (Fig. 6D). TUNEL staining indicated that the addition of compound C treatment eliminated the decrease in the number of TUNEL-positive cells caused by the canagliflozin treatment in the kidneys of cisplatin-treated mice (Fig. 6E, F). Canagliflozin treatment diminished (did not diminish) the expression of cleaved caspase-3 in the kidneys of cisplatin-treated mice without (with) compound C administration (Fig. 6G, H).

## Discussion

SGLT2 inhibitors have received attention in the field of diabetes mellitus management because of their beneficial impact on the cardiovascular and renal outcomes in patients with diabetes mellitus [1–6]. This class of drugs has beneficial effects even on patients without diabetes mellitus and several animal models of non-diabetic kidney diseases [28]. However, available data are limited, some studies showed inconsistent results, and preclinical studies mainly focused on the animal models of chronic kidney disease. Furthermore, these studies rarely illustrated the underlying mechanism of the beneficial effects rendered by SGLT2 inhibitors.

In this study, we showed that canagliflozin protects HK-2 cells from cisplatin-induced cytotoxicity and inhibits the expression of various apoptosis markers upon cisplatin treatment in a concentration-dependent manner. Recently, several studies have suggested that SGLT2 inhibitors protect against various non-diabetic disease models [10, 11, 29, 30]. However, there are few studies covering the effect of SGLT2 inhibitors directly on cell death. *Lieberthal* et al. [31]. revealed that lower concentrations of cisplatin (micromolar) mainly induce apoptosis of renal tubular cells, whereas high concentrations of cisplatin (millimolar) lead to the necrosis of the cells. Collectively, our data suggest that canagliflozin attenuates cisplatin-induced cytotoxicity by inhibiting apoptosis in the HK-2 cells.

We observed that canagliflozin increases autophagic flux in HK-2 cells in a concentration-dependent manner and augments autophagic flux in cisplatin-treated HK-2 cells. Moreover, canagliflozin protects against cisplatin-induced cytotoxicity in an autophagy-dependent manner. The pro-survival function of autophagy has been demonstrated in various kidney disease models involving cell death of proximal tubular epithelium, such as ischemia-reperfusion injury, sepsis-induced AKI, cyclosporin nephrotoxicity, and cisplatin-induced AKI [19–22]. Many studies showed the possibility that SGLT2 inhibitors improve or restore autophagy in diabetic disease models;[32–35] thus, autophagy is considered an underlying mechanism for cardioprotective and renoprotective effects in patients with diabetes mellitus using SGLT2 inhibitors [24, 25]. However, few studies support the possibility that SGLT2 inhibitors induce autophagy in non-diabetic disease models [36, 37]. Our data suggest that canagliflozin increases autophagic flux in non-diabetic conditions and protects HK-2 cells from cisplatin-induced apoptosis in an autophagy-dependent manner. Since the mitochondria-associated proteins have been suggested as mediators for cross-talk between apoptosis and autophagy and accumulating evidence suggests that mitophagy has a protective effect in the various forms of AKI including cisplatin-induced AKI, the protective effect of canagliflozin might be associated with the mitophagy [38–41]. These findings also support the existing evidence that SGLT2 inhibitors can induce autophagy and be applied to pathological conditions other than diabetes mellitus.

We verified the involvement of the AMPK-mTOR pathway, a major signaling pathway that regulates autophagy, in the protective effect of canagliflozin against cisplatin-induced cytotoxicity in HK-2 cells. Canagliflozin induces phosphorylation of AMPK and subsequent dephosphorylation of mTOR and provides protection in an AMPK activation-dependent manner. SGLT2 inhibitors augment AMPK phosphorylation in diverse circumstances [37, 42, 43]. Several studies on cell culture models demonstrated that only canagliflozin, not dapagliflozin or empagliflozin, activates AMPK via the inhibition of mitochondrial respiratory complex in concentrations corresponding to the peak plasma concentrations achieved by administration of therapeutic doses in humans [44, 45]. In line with these results, our findings showed that only canagliflozin reduced the cytotoxicity of cisplatin in HK-2 cells. Canagliflozin has features different from those of dapagliflozin or empagliflozin that activate AMPK and enable canagliflozin to protect against cisplatin-induced cytotoxicity in HK-2 cells.

Since the kidney is a highly complex organ composed of >20 different cell types and its microenvironment plays an important role in injury and repair processes, in vitro assays cannot adequately reflect the disease process and outcome [46]. Therefore, we conducted animal experiments using the mouse model of cisplatin-induced AKI. In animal experiments, canagliflozin attenuated the cisplatin-induced tubular damage assessed by histopathology and immunoblot analysis of kidney lysates. Furthermore, canagliflozin also ameliorated the deterioration of kidney function induced by cisplatin administration. Immunohistochemistry staining of LC3B indicated that canagliflozin increased autophagy in proximal tubular cells of mouse kidneys. These results agree with the results from in vitro experiments. To the best of our knowledge, this study is the first report demonstrating the possibility that SGLT2 inhibitors can induce autophagy in non-diabetic or metabolically normal mouse kidneys whereas previous studies on SGLT2 inhibitors and autophagy mainly focused on the mouse models of diabetes or obesity [34, 47]. Existing data and our results from in vitro experiments suggest that canagliflozin has unique features that directly activate AMPK and induce autophagy in the cells. In addition to this mechanism, ketogenesis elicited by the administration of SGLT2 inhibitors can be an underlying mechanism that induces autophagy at the animal level [48, 49].

Results from the animal experiments with chloroquine or compound C indicate that the protective effect of canagliflozin depends on autophagy induced by AMPK activation in the mouse model of cisplatin-induced AKI. These results correspond to those of in vitro experiments and replicate the involvement of autophagy and AMPK pathway in the cisplatin-induced AKI illustrated in previous studies [50, 51].

SGLT2 inhibitors have clinical benefits in patients even without diabetes mellitus. This aspect has inspired many clinicians or researchers to discover other implications of SGLT2 inhibitors in various diseases other than diabetes mellitus. In this study, canagliflozin treatment protected the renal proximal tubular cells from cisplatin treatment and thereby attenuated the development of AKI in mice. Canagliflozin-induced activation of AMPK and the following induction of autophagy plays a critical role in this protective role. This study provides evidence that treatment with canagliflozin may protect against AKI caused by proximal tubular injury.

## Materials and methods

### Cell culture and treatments

HK-2 cells (ATCC, Manassas, VA, USA), which were immortalized by transduction with human papillomavirus 16 E6/E7 genes, were maintained in a keratinocyte serum-free medium (Thermo Fisher Scientific, Waltham, MA, USA) supplemented with 5 ng/mL human recombinant epidermal growth factor 1–53 and 50 μg/mL bovine pituitary extract at 37 °C in a humidified 5% CO_2_.

For analyzing the protective role of SGLT2 inhibitors in cisplatin-induced cytotoxicity, HK-2 cells were seeded in 6-well or 96-well plates and treated with 20 μM cisplatin (Sigma-Aldrich, St. Louis, MO, USA) in the absence or presence of 1–25 μM canagliflozin, dapagliflozin, or empagliflozin (APExBIO, Houston, TX, USA) for 24 h.

To test whether canagliflozin induces autophagy or modulates AMPK-mTOR pathway and whether the protective effect of canagliflozin on cisplatin-induced cytotoxicity occurs in autophagy or AMPK activation-dependent manner, the cells were seeded in 6-well or 96-well plates and treated with 20 μM cisplatin in the absence or presence of 10 μM canagliflozin and/or 10 μM chloroquine, 5 nM bafilomycin A (Sigma-Aldrich), or 1 μM compound C (APExBIO) for 24 h.

### Animal experiments

The animal experiments were approved by and performed in accordance with the Institutional Animal Care and Use Committee of Yonsei University Health System (2020–0128). Male C57BL/6 mice (8–10-week-old) were obtained from Orient Bio (Seongnam, Korea) and housed in a specific pathogen-free facility with a 12 h:12 h light–dark cycle and fed with a standard diet and tap water at controlled room temperature (RT, 22 ± 1 °C) and humidity (50 ± 5%) in the animal facility of Yonsei University. The doses of the chemicals used in the experiments were determined based on previously published studies [50–52]. For cisplatin treatment, mice were injected with a single dose of cisplatin (20 mg/kg, i.p.), while control animals were injected with a comparable volume of saline. To test the effect of canagliflozin, canagliflozin dissolved in 0.1% carboxymethyl cellulose (10 mg/kg/d) or a vehicle solution was administered via oral gavage for five consecutive days before cisplatin treatment. To test the effect of chloroquine or compound C, 60 mg/kg/d, i.p chloroquine or 5 mg/kg/d, i.p. compound C was injected for five consecutive days before cisplatin treatment and control animals were injected with a comparable volume of saline. Animals were euthanized 72 h after cisplatin treatment to collect blood samples and kidneys for analysis.

### Cell viability assay

A cell counting kit-8 (CCK-8) (Dojindo Molecular Technologies, Rockville, MD, USA) assay was used to measure the cytotoxicity of cisplatin on HK-2 cells. HK-2 cells were seeded in 96-well plates and treated with cisplatin in the absence or presence of canagliflozin, and/or an autophagy inhibitor (chloroquine or bafilomycin A) or an AMPK inhibitor (compound C). After 24 h, the CCK-8 solution (10 μL) was added to each well, followed by incubation for 2 h at 37 °C. The absorbance at 450 nm was measured using a microplate reader. Cell viability was expressed as a percentage of the control.

### Measurement of renal function

Blood samples were collected from a cardiac puncture at the time of euthanasia using a serum separation tube (Becton, Dickinson and Company, Franklin Lakes, NJ, USA). Serum was collected by centrifugation after clotting at RT. Renal function was determined by measuring BUN and serum creatinine using commercial kits (Fujifilm, Tokyo, Japan).

### Histological examination

Kidneys were perfused with saline, harvested, and then fixed with 4% paraformaldehyde overnight at 4 °C for paraffin embedding. The paraffin-embedded tissues were sectioned into 4 μm thickness and stained with hematoxylin and eosin (H–E). Tubular damage was indicated by the loss of the brush border, tubular dilation, cast formation, and cell lysis. The tubular injury score was obtained in a blind manner and scored by the percentage of damaged tubules as follows: 0, no damage; 1, <25%; 2, 25–50%; 3, 50–75%; 4, 75–90%; 5, ≥90%. The score was reported as the mean of 10 random high-power fields (×400) per section.

### Examination of apoptosis

Apoptosis in the renal tissue was detected via TUNEL assay using the TACS2^®^ TdT DAB (diaminobenzidine) kit (Trevigen, Inc., Gaithersburg, MD, USA) according to the manufacturer’s instructions. For the quantification of the TUNEL-positive cells, 10 high-power fields (×400) were randomly selected from each section and the number of TUNEL-positive cells was counted.

### Immunohistochemical staining of LC3B

Paraffin-embedded kidney sections of 4 μm thickness were deparaffinized and then subjected to antigen retrieval in 1 mM EDTA and 0.05% Tween-20 and at pH 8.0 and 96–100 °C for 1 h. The sections were then exposed to 3% H_2_O_2_ for 10 min and then to the buffer solution containing 2% bovine serum albumin, 0.2% milk, 2% normal goat serum, and 0.8% Triton X-100 and anti-LC3B antibody (NB100-2220, Novus Biologicals, Littleton, CO, USA) at RT for 1 h. Then, the sections were incubated with the EnVision+ HRP goat anti-rabbit antibody (K4003, Dako, Glostrup, Denmark) for 1 h at RT. After further washing, the signals were developed using a DAB kit (Dako). The amplitude of LC3B staining was reported as the mean of 10 random high-power fields (×400) per section using ImageJ.

### Immunoblot analysis

Whole cells or freshly frozen tissues were lysed in a radioimmunoprecipitation assay buffer (GenDEPOT, Katy, TX, USA) containing a protease inhibitor cocktail (GenDEPOT) and phosphatase inhibitor cocktail (Thermo Fisher Scientific). Protein concentration was estimated using a BCA protein assay kit (Thermo Fisher Scientific). Equal amounts of protein were loaded in each lane and separated by SDS-PAGE. After being transferred to nitrocellulose membranes, membranes were blocked in 5% milk, probed with primary antibodies (anti-cleaved caspase-3 (#9661), anti-PARP, anti-total-AMPK (#2532), anti-phospho-mTOR (#2971), anti-total-mTOR (#2983), and anti-β-actin (#4967) (Cell Signaling Technology, Danvers, MA, USA), anti-γ-H2AX (ab11174), anti-phospho-AMPK (ab133448) (Abcam, Cambridge, UK), and anti-LC3B (L7543, Sigma-Aldrich)), probed with the appropriate secondary antibodies, and visualized by using an enhanced chemiluminescence kit.

### Annexin V/Propidium iodide (PI) staining and flow cytometric analysis

Briefly, HK-2 cells were treated as aforementioned. Then, the cells were harvested and stained with annexin V-FITC/PI staining kit (Becton, Dickinson and Company) according to the manufacturer’s instruction and analyzed by flow cytometry. Annexin V positive cells were considered apoptotic cells.

### Statistical analysis

For immunoblot analysis, films were scanned, and the band density was determined using a densitometer and the image analysis program, ImageJ. The experimental data were analyzed by performing the Student’s *t*-test or one-way analysis of variance (ANOVA) test using GraphPad Prism. The results represent the mean value and SD, as indicated in the figure legends. The difference was considered statistically significant when *p* < 0.05.

## Supplementary information

Supplementary Figure Legends

Supplementary Figure 1

Supplementary Figure 2

Supplementary Figure 3

Supplementary Figure 4

Supplementary Figure 5

Declaration of contribution to article

## Data Availability

All data generated or analyzed during this study are included in this published article and its supplementary information files.
