# Supplementary material for: Canagliflozin protects against cisplatin-induced acute kidney injury by AMPK-mediated autophagy in renal proximal tubular cells
Source: Cell Death Discov. 2022 Jan 10;8:12. doi: 10.1038/s41420-021-00801-9 (PMC8748642; doi:10.1038/s41420-021-00801-9)
Supplement: Supplementary file 1 — Supplementary Figure Legends [file 41420_2021_801_MOESM1_ESM.docx]

**Supplementary Figure Legends**

**Supplementary Figure 1**

Cell viability was evaluated via CCK-8 assay. Data are expressed as mean±SD. Differences between groups were evaluated using one-way ANOVA followed by Bonferroni’s multiple comparison test. **P<0.01, ***P<0.001, significantly different from control. NS, not significant. (A) HK-2 cells were treated with indicated concentrations of cisplatin for 24 h (N=4). (B) HK-2 cells were treated with 20 μM cisplatin for the indicated durations (N=3–4). (C) HK-2 cells were treated with indicated concentrations of canagliflozin (CANA) for 24 h (N=5). (D–E) HK-2 cells were treated with 20 μM cisplatin in the absence or presence of dapagliflozin (DAPA) (D) or empagliflozin (EMPA) (E) at the indicated concentrations for 24 h (N=8).

**Supplementary Figure 2**

(A) HK-2 cells were treated with the indicated concentrations of canagliflozin (CANA) for 24 h. (B) HK-2 cells were treated with indicated concentrations of cisplatin and/or canagliflozin for 24 h. (C) HK-2 cells were treated with indicated concentrations of canagliflozin and/or chloroquine (CQ) for 24 h (N=5). Cell viability was evaluated via CCK-8 assay. Data are expressed as mean±SD. Differences between groups were evaluated using one-way ANOVA followed by Bonferroni’s multiple comparison test. NS, not significant. HK-2 cells were treated with indicated concentrations of cisplatin, canagliflozin, and/or chloroquine for 24 h (D–F). (D) The representative graphs showing the results of Annexin V/PI staining analyzed by flow cytometry. (E) The graph showing the proportion of apoptotic cells from Annexin V/PI staining. Data are expressed as mean±SD (N=3). Differences between groups were evaluated using one-way ANOVA followed by Bonferroni’s multiple comparison test. **P<0.01. NS, not significant. (F) Representative immunoblot analysis containing non-cleaved caspase-3. (G) HK-2 cells were treated with indicated concentrations of canagliflozin and/or bafilomycin A (Baf A) for 24 h (N=5). Cell viability was evaluated via CCK-8 assay. Data are expressed as mean±SD. Differences between groups were evaluated using one-way ANOVA followed by Bonferroni’s multiple comparison test. NS, not significant. HK-2 cells were treated with indicated concentrations of cisplatin, canagliflozin, and/or bafilomycin A for 24 h (H–J). (H) Cell viability was evaluated via CCK-8 assay. Data are expressed as mean±SD (N=6). Differences between groups were evaluated using one-way ANOVA followed by Bonferroni’s multiple comparison test. ***P<0.001. NS, not significant. (I) Representative immunoblot analysis. (J) Densitometric analysis of immunoblots to estimate the relative abundance of cleaved caspase-3 as normalized that of β-actin. Data are expressed as mean±SD (N=3). Differences between groups were evaluated using one-way ANOVA followed by Bonferroni’s multiple comparison test. ***P<0.001. NS, not significant.

**Supplementary Figure 3**

HK-2 cells were treated with indicated concentrations of canagliflozin (CANA) and/or compound C (Comp. C) for 24 h. Cell viability was evaluated via CCK-8 assay. Data are expressed as mean±SD (N=6). Differences between groups were evaluated using one-way ANOVA followed by Bonferroni’s multiple comparison test. NS, not significant.

**Supplementary Figure 4**

C57BL/6 mice were administered with canagliflozin (CANA) (10 mg/kg) or vehicle orally and intraperitoneally injected with chloroquine (CQ) (60 mg/kg) or saline for five consecutive days. Mice were euthanized at 72 h after the last canagliflozin and chloroquine treatment to collect blood samples for measurements of blood urea nitrogen and serum creatinine and kidney tissues for histology. (A) Representative images of kidney H–E staining (scale bar = 100 μm). (B) Blood urea nitrogen. (C) Serum creatinine. Data are expressed as mean±SD (N=3). Differences between groups were evaluated using Student’s paired *t*-test. NS, not significant.

**Supplementary Figure 5**

C57BL/6 mice were administered with canagliflozin (CANA) (10 mg/kg) or vehicle orally and intraperitoneally injected with compound C (Comp. C) (5 mg/kg) or saline for five consecutive days. Mice were euthanized at 72 h after the last canagliflozin and compound C treatment to collect blood samples for measurements of blood urea nitrogen and serum creatinine and kidney tissues for histology. (A) Representative images of kidney H–E staining (scale bar = 100 μm). (B) Blood urea nitrogen. (C) Serum creatinine. Data are expressed as mean±SD (N=3). Differences between groups were evaluated using Student’s paired *t*-test. NS, not significant.
