# Supplementary figures and images for: Canagliflozin protects against cisplatin-induced acute kidney injury by AMPK-mediated autophagy in renal proximal tubular cells
Source: Cell Death Discov. 2022 Jan 10;8:12. doi: 10.1038/s41420-021-00801-9 (PMC8748642; doi:10.1038/s41420-021-00801-9)

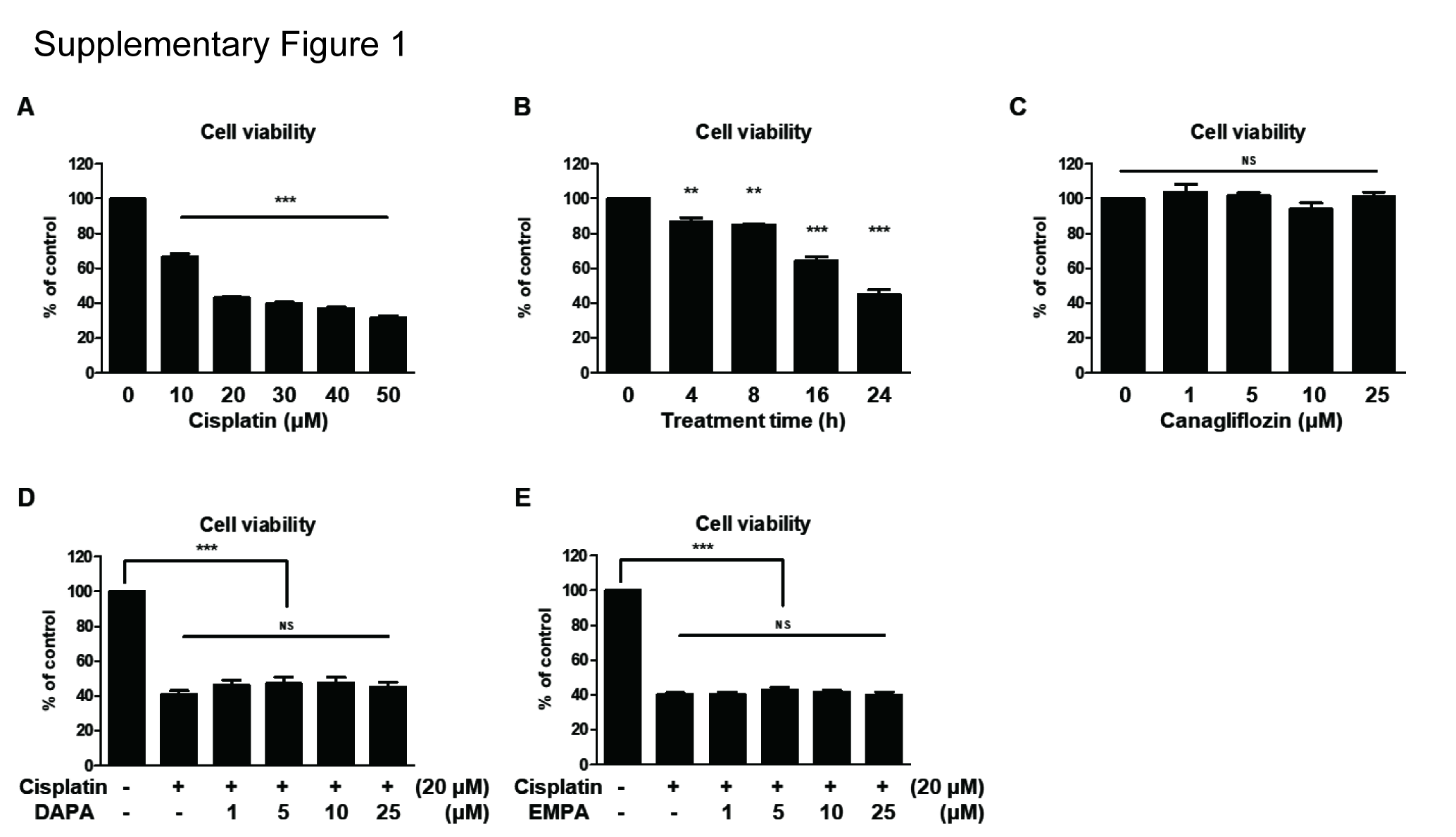

Supplement: Supplementary file 2 — Supplementary Figure 1 [file 41420_2021_801_MOESM2_ESM.tif]

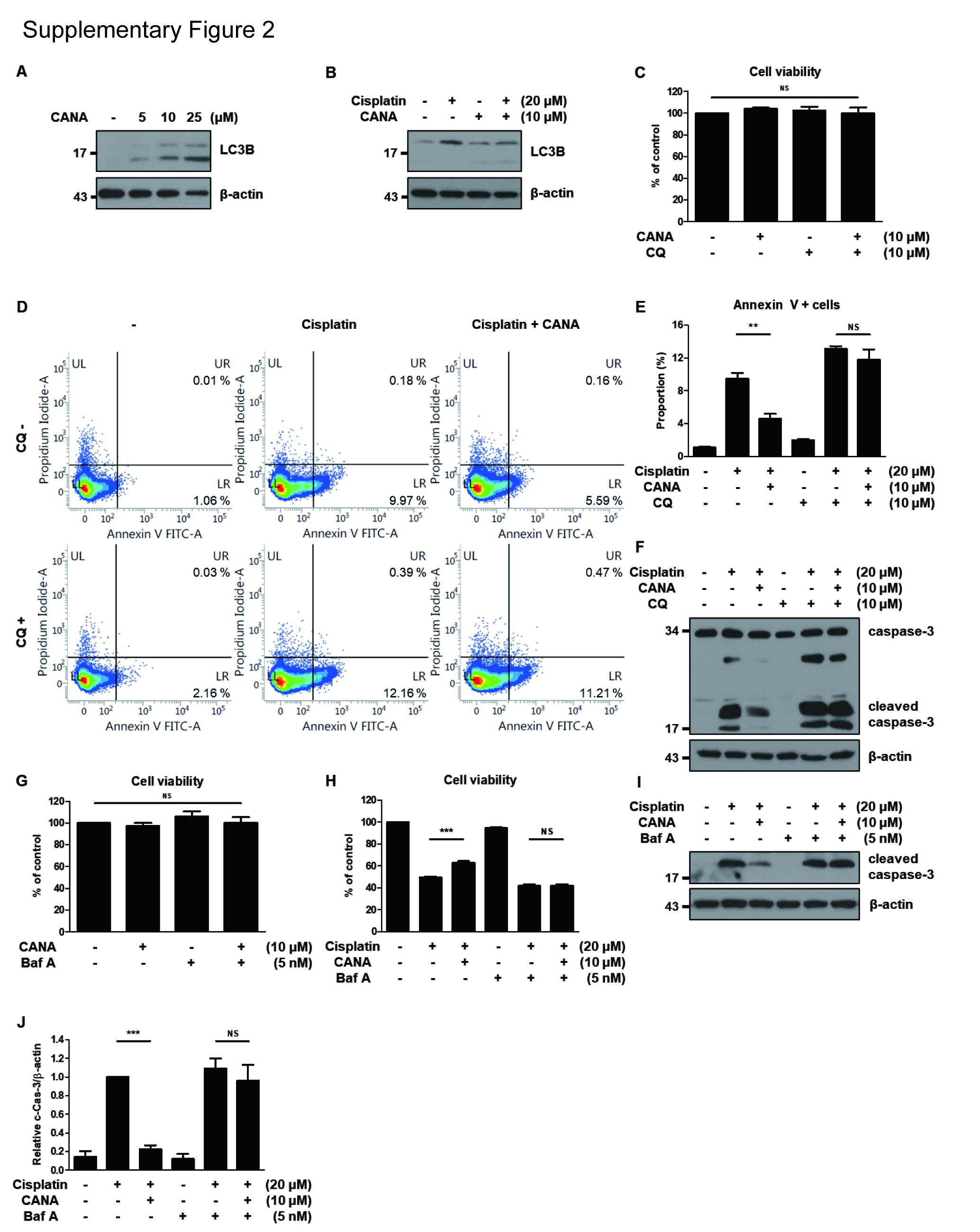

Supplement: Supplementary file 3 — Supplementary Figure 2 [file 41420_2021_801_MOESM3_ESM.tif]

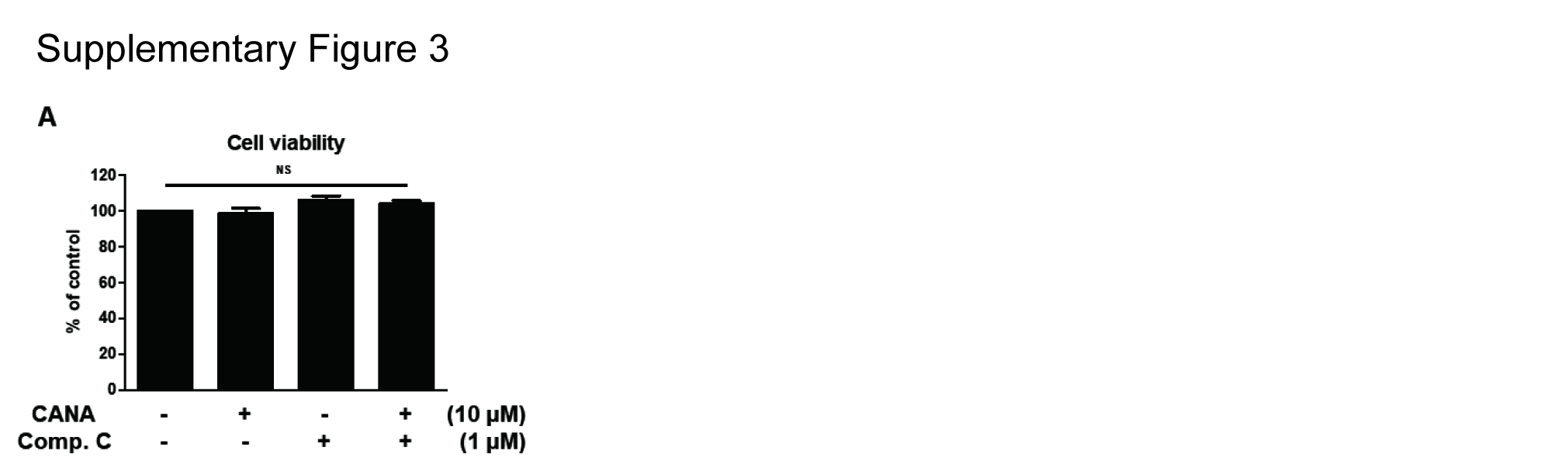

Supplement: Supplementary file 4 — Supplementary Figure 3 [file 41420_2021_801_MOESM4_ESM.tif]

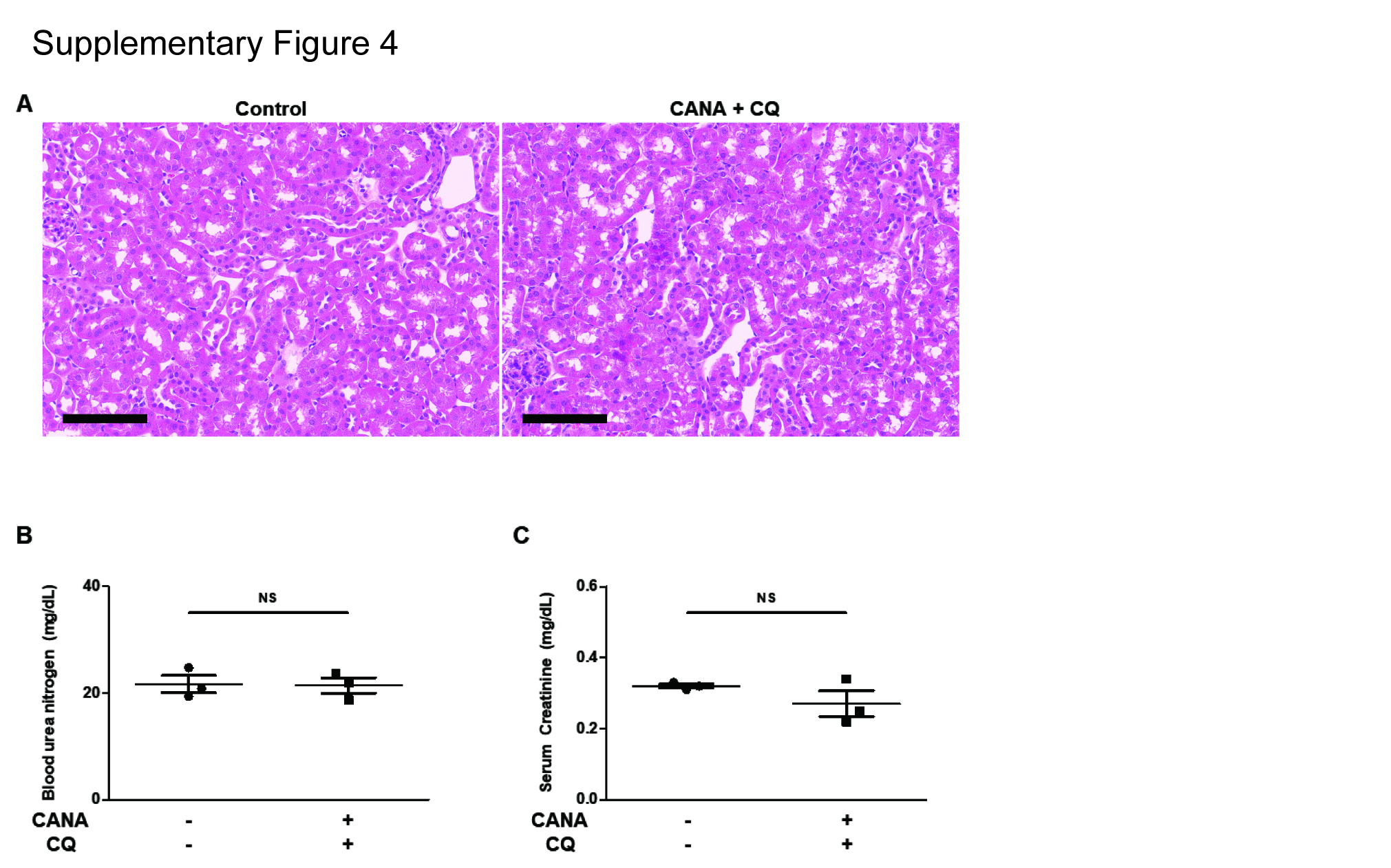

Supplement: Supplementary file 5 — Supplementary Figure 4 [file 41420_2021_801_MOESM5_ESM.tif]

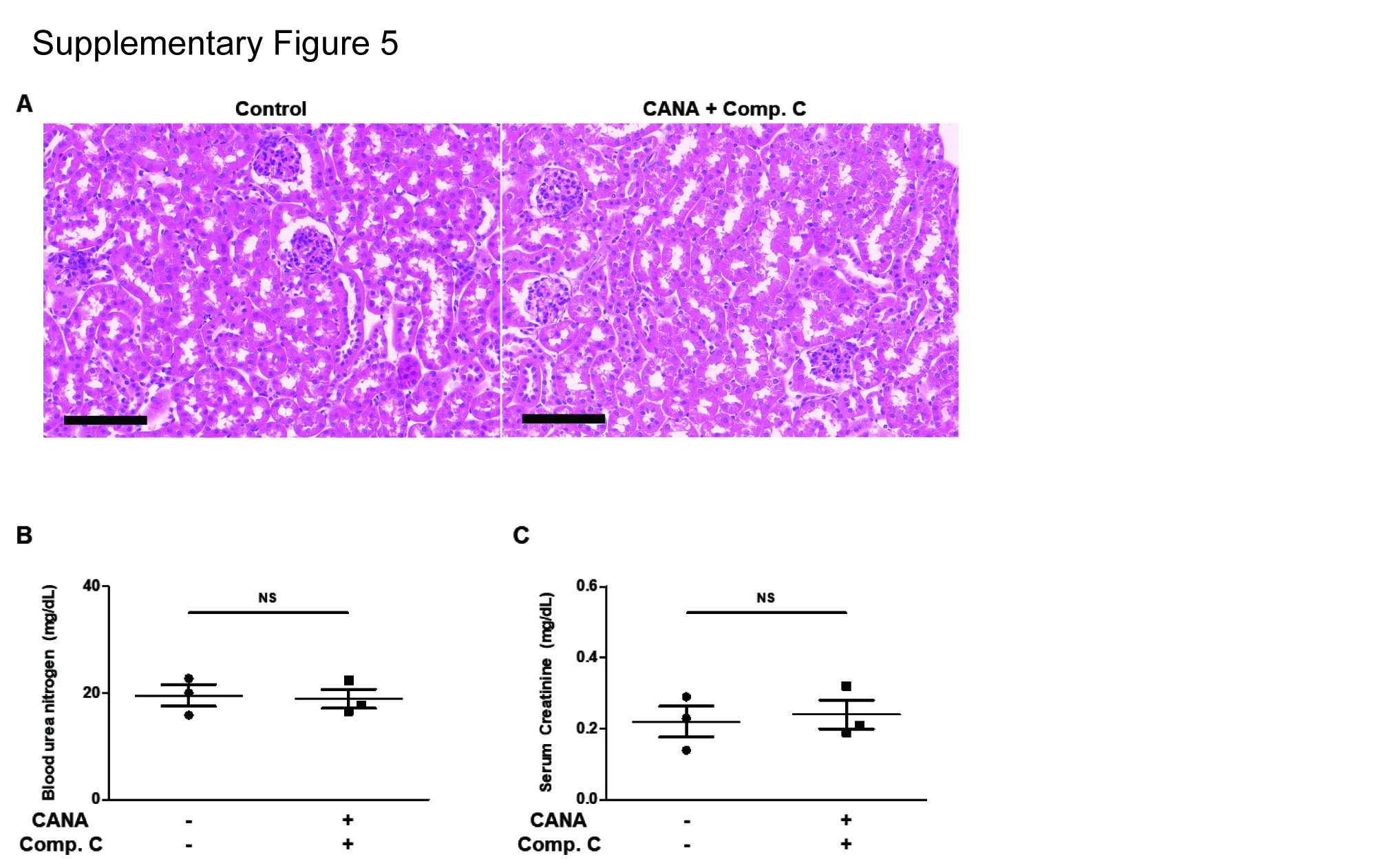

Supplement: Supplementary file 6 — Supplementary Figure 5 [file 41420_2021_801_MOESM6_ESM.tif]
