# Supplementary material for: Canagliflozin protects against cisplatin-induced acute kidney injury by AMPK-mediated autophagy in renal proximal tubular cells
Source: Cell Death Discov. 2022 Jan 10;8:12. doi: 10.1038/s41420-021-00801-9 (PMC8748642; doi:10.1038/s41420-021-00801-9)
Supplement: Supplementary file 7 — Declaration of contribution to article [file 41420_2021_801_MOESM7_ESM.pdf]

DECLARATION OF CONTRIBUTIONS TO ARTICLE

ADMC

Manuscript Number: CDDISCOVERY-21-2991R1

Journal Name: *Cell Death Discovery* (the 'Journal')

Proposed Title of the Contribution:

Canagliflozin protects against cisplatin-induced acute kidney injury by AMPK-mediated autophagy in renal proximal tubular cells (the 'Contribution')

Author(s):

Cheol Ho Park, Bin Lee, Myeong Gil Han, Woo Joong Rhee, Man Sup Kwak, Tae-Hyun Yoo, and Jeon-Soo Shin (the 'Authors')

For all *CDDis* articles, each person named as an author in the published version must be able to show he or she has contributed substantially to the article.

Authorship credit should be based on 1) substantial contributions to conception and design, acquisition of data, or analysis and interpretation of data; 2) drafting the article or revising it critically for important intellectual content; and 3) final approval of the version to be published. Authors should meet conditions 1, 2 and 3.

Any person who cannot be shown to have made a substantial contribution to the article cannot be listed as an author in the final version. The name of any person who is deemed to have made a minor contribution can, however, appear in the Acknowledgments section of the article.

Please complete the table below to indicate the contributions of all named authors to the manuscript.

Author Full Name:	Specification of Contribution to the Manuscript:
Cheol Ho Park	Conception, design, acquisition of data, analysis and interpretation of data, and drafting the article
Bin Lee	Acquisition of data and analysis and interpretation of data
Myeong Gil Han	Analysis and interpretation of data
Woo Joong Rhee	Acquisition of data, analysis and interpretation of data
Man Sup Kwak	Conception, design, and analysis and interpretation of data
Tae-Hyun Yoo	Conception, design, and revising the article
Jeon-Soo Shin	Conception, design, revising the article, and final approval of the version to be published

Please complete the table below to indicate the contributions of all named authors to the figures.

Figure 1:

CHP generated and prepared panel A and B.
CHP and BL generated and prepared panel C–E.
CHP assembled the figure.

Figure 2:

CHP and BL generated and prepared panel A–D.
CHP generated and prepared panel E and F.
CHP and BL generated and prepared panel G.
CHP assembled the figure.

Figure 3:

CHP generated and prepared panel A–F.
CHP assembled the figure.

Figure 4:

CHP conducted the animal experiments.
CHP generated and prepared panel A–C.
CHP and WJR scored tubular injury indicated in H–E stain result in panel D.
CHP prepared panel D and E.
CHP and MGH counted the number of TUNEL positive cells indicated in TUNEL assay of panel F.
CHP prepared panel F.
CHP generated and prepared panel G–I.
CHP and MSK quantified the intensity of LC3B staining on the immunohistochemical staining in panel J.
CHP prepared panel J.
CHP generated and prepared panel K and L.
CHP assembled the figure.

Figure 5:

CHP conducted the animal experiments.
CHP generated and prepared panel A–C.
CHP and WJR scored tubular injury indicated in H–E stain result in panel D.
CHP prepared panel D and E.
CHP and MGH counted the number of TUNEL positive cells indicated in TUNEL assay of panel F.
CHP prepared panel F.
CHP generated and prepared panel G and H.
CHP assembled the figure.

Figure 6:

CHP conducted the animal experiments.
CHP generated and prepared panel A–C.
CHP and WJR scored tubular injury indicated in H–E stain result in panel D.
CHP prepared panel D and E.
CHP and MGH counted the number of TUNEL positive cells indicated in TUNEL assay of panel F.
CHP prepared panel F.
CHP generated and prepared panel G and H.
CHP assembled the figure.

Signed for and on behalf of the Author(s):	Print Name:	Date:
J. S. Shin	Jeon-Soo Shin	November 23, 2021
